# Building a Framework for Care of Older Patients in an Academic Setting: High Risk Geriatrics Ambulatory Care Program

**DOI:** 10.1093/geroni/igaa057.1684

**Published:** 2020-12-16

**Authors:** Stephanie Chow, Audrey Chun, Lizette Munoz, Shamsi Fani, Rolfe Sean Morrison, Blair MacKenzie, Susana Lavayen, William Hung

**Affiliations:** 1 Icahn School of Medicine at Mount Sinai, New York, New York, United States; 2 Icahn School of Medicine at Mount Sinai, New york, New York, United States; 3 Mount Sinai Hospital, New York, New York, United States; 4 Mount Sinai Medical Center, New York, New York, United States; 5 Icahn School of Medicine at Mount Sinai; James J Peters VA Medical Center, Bronx, New York, United States

## Abstract

BACKGROUND: Traditional models of geriatric medicine and health system reimbursement structure often force ambulatory care teams to function as high-volume delivery programs, thereby dis-servicing our most vulnerable and frail older patients. This “high cost and high needs” labeled demographic requires uniquely adapted plans from medical and social work providers. METHODS: To better examine opportunities for improved framework for geriatric ambulatory care, the Acute Life Interventions, Goals & Needs (ALIGN) Program has launched several inter-professional pilot programs, each with intention to explore components of health care service to older patients, and feasibility of implementation in other health care systems. Three current models include the ALIGN Program itself, a telemedicine community paramedicine program, and a geriatric surgery co-management program. RESULTS: Preliminary results are forthcoming, with initial promising findings. For the first 126 patients enrolled, mean emergency room (ED) visits 6 months prior to ALIGN enrollment were 1.7 visits per person, reduced to 0.7 ED visits/person 6 months post-graduation from the program, and 126 fewer ED visits. Mean hospitalization 6 months prior to enrollment was 0.32 per person, whereas 6 months post-graduation was 0.2 hospitalizations/person, totaling 40.32 hospitalizations saved. Mean length of stay in the hospital 6 months prior to ALIGN enrollment for the 22 patients admitted was 7.7 days, reduced to 7.3 days post-graduation, and 32 fewer hospital days in the small subset of patients requiring hospitalization despite program interventions. CONCLUSION: The ALIGN Program’s multi-professional and flexible modularity highlights promising innovative frameworks for ambulatory geriatrics care, warranting further exploration and collaboration.

